# Kinesin family member 11 is a potential therapeutic target and is suppressed by microRNA‐30a in breast cancer

**DOI:** 10.1002/mc.23203

**Published:** 2020-04-29

**Authors:** Benfang Wang, Jianjiang Yu, Zhenjiang Sun, Frank Luh, Dandan Lin, Ying Shen, Ting Wang, Qi Zhang, Xiyong Liu

**Affiliations:** ^1^ Department of Clinical Laboratory Jiangyin People's Hospital Affiliated to Nantong University Jiangyin China; ^2^ MOH Key Lab of Thrombosis and Hemostasis, Collaborative Innovation Center of Hematology‐Thrombosis and Hemostasis Group, Jiangsu Institute of Hematology, The First Affiliated Hospital of Soochow University, Soochow University Suzhou China; ^3^ Sino‐American Cancer Foundation Temple City California; ^4^ Department of Chinese‐Western Medicine Integrative Oncology The First Affiliated Hospital of Anhui Medical University Hefei China; ^5^ School of Medicine, Zhejiang University City College Hangzhou Zhejiang China; ^6^ Department of Tumor Biomarker Development California Cancer Institute Temple City California

**Keywords:** breast cancer, kinesin family member 11, microRNA‐30a, prognostic biomarker, therapeutic target

## Abstract

Kinesin family member 11 (KIF11) is a plus end‐directed kinesin indispensable for the formation of the bipolar spindle in metaphase, where it objects to the action of minus end‐directed molecular motors. Here, we hypothesize that *KIF11* might be a therapeutic target of breast cancer and regulated by *miR‐30a*. Cell Counting Kit 8 assays were used to investigate cell proliferation. Invasion assays were used to survey the motility of cells. Kaplan‐Meier and Cox proportional analyses were employed for this outcome study. The prognostic significance and performance of *KIF11* were validated on 17 worldwide independent microarray datasets and two The Cancer Genome Atlas‐Breast Invasive Carcinoma sets. microRNA was predicted targeting *KIF11* through sequence alignment in microRNA.org and confirmed by coexpression analysis in human breast cancer samples. Dual‐luciferase reporter assays were employed to validate the interaction between *miR‐30a* and *KIF11* further. Higher *KIF11* mRNA levels and lower *miR‐30a* were significantly associated with poor survival of breast cancer patients. Inhibition of *KIF11* by small‐hairpin RNA significantly reduced the proliferation and invasion capabilities of the breast cancer cells. Meanwhile, downregulation of *KIF11* could enhance the cytotoxicity of adriamycin in breast cancer cell lines MCF‐7 and MDA‐MB‐231. A population study also validated that chemotherapy and radiotherapy significantly improved survival in early‐stage breast cancer patients with low *KIF11* expression levels. Further bioinformatics analysis demonstrated that *miR‐30a* could interact with *KIF11* and validated by dual‐luciferase reporter assays. Therefore, *KIF11* is a potential therapeutic target of breast cancer. *miR‐30a* could specifically interact with *KIF11* and suppress its expression in breast cancer.

## 1 INTRODUCTION

Breast cancer is one kind of the most common malignant cancers among females in the world, with approximately 1 000 000 new cases each year.^1^, ^2^ It is also the second‐leading cause of death among women, accounts for 15% of all cancer deaths.^3^ Multiple oncogenes, tumor suppressor genes, sex steroid hormones, and their receptors are involved in the genesis and development of breast cancer. Breast cancer is a heterogeneous tumor. There are a variety of subtypes with different biological behaviors and clinicopathologic features that can result in obviously different prognoses, which can be divided into four major molecular subtypes: luminal A (LumA), luminal B (LumB), triple‐negative/basal‐like, and human epidermal growth factor receptor 2 (HER2) type.^4^, ^5^, ^6^, ^7^, ^8^ This classification of breast cancers has been used for selecting the appropriate therapeutic method. Currently, personalized precision medicine is an emerging field, however, underdeveloped in breast cancer. More targets and corresponding inhibitors need to be explored to improve treatment efficacy and to reduce adverse side effects.

The protein of the kinesin family could function as molecular nanomotors. It converts the free energy of nucleotide hydrolysis in coordinating the mechanical movement of microtubules.^9^, ^10^ As a member of the kinesin family, KIF11 is a microtubule‐dependent motor protein encoded by the *KIF11* gene located at 10q24.1, with a primary function in mitotic spindle formation.^11^ KIF11 is still an essential element for maintaining proper spindle dynamics and preserving spindle bipolarity in cell division. It has a catalytic motor/ATPase domain that mediates its interaction with ATP and microtubules. KIF11 utilizes the energy released by ATP hydrolysis to move forward along microtubules. It facilitates spindle assembly by forming a homotetramer. The homotetramer can cross‐link and push apart antiparallel microtubules.^12^, ^13^ In the previous study, the *KIF11* has been implicated in tumourigenesis. It overexpresses in blast crisis chronic myeloid leukemia, activation in mouse B‐cell leukemia, and triggering of genomic instability in transgenic mice.^14^, ^15^, ^16^ *KIF11* has also been identified as a molecule involved in pancreatic cancer, non–muscle invasive bladder urothelial carcinoma, non–small cell lung cancer, and glioblastoma.^17^, ^18^, ^19^, ^20^ These studies suggest that *KIF11* may be involved in the pathogenesis of multiple kinds of cancer. Because of its participating in dividing cells, *KIF11* is an essential anticancer target with the trait to avoid the deficiencies of traditional anti‐mitosis drugs.^21^, ^22^ Drug candidates like ispinesib inhibit *KIF11* and cause mitotic arrest, then apoptosis. The research and development of ligand are continually driven partly by the observation of deactivating mutations in the drug binding region, and lack of successful monotherapies based on *KIF11* inhibition. Although in the course of our research, one study has discussed the function of *KIF11* in breast cancer,^23^ whether *KIF11* is a potential therapeutic target for breast cancer remains unclarified currently, and the transcriptional regulation on *KIF11* also needs to be elucidated.

As an essential transcriptional regulator, the differential expression pattern of microRNAs (miRNAs) in health and disease, therapeutic response, and resistance has resulted in its application as robust biomarkers.^24^ Gene regulation by miRNAs and reciprocal regulation of miRNAs have now been studied for over 15 years and extensively reviewed.^25^ In general, one miRNA could target multiple genes. Meanwhile, one messenger RNA (mRNA) can be targeted by multiple miRNAs, which highlighted the complexity of miRNA biology.^26^ Previous studies showed that the outcome of cancer is closely related to the variable expression and the specific expression signatures of miRNA in cancer tissues.^27^ In particular, there is some existing evidence that miRNAs are tightly linked to the development of human breast cancer.^28^, ^29^, ^30^, ^31^ miRNAs are attractive candidates as upstream regulators of breast tumor progression and metastasis by regulating entire sets of genes. miRNA signature can subclassify breast cancer^32^ and can even determine new subtypes, as recently reported.^33^ *miR‐30a* has been validated as a tumor suppressor via targeting multiple genes in diverse cancer.^34^, ^35^, ^36^, ^37^ Here, we predicted target miRNAs of *KIF11* using both predicting *KIF11*‐related miRNAs in microRNA.org and correlation analysis for *KIF11* and miRNAs in the GSE22220 dataset, screening out target‐*KIF11* miRNAs. Favorably, *miR‐30a* was one of five miRNAs that could bind to the 3'‐untranslated region (3'‐UTR) of *KIF11* mRNA. Thus, *miR‐30a* may be involved in the regulation of *KIF11* in cancer progression.

In the present study, we investigated the role of *KIF11* in the tumorigenesis and treatment of breast cancer and the possible role of *miR‐30a* in the regulation of *KIF11* in this process.

## 2 MATERIALS AND METHODS

### 2.1 Cell culture and chemicals

Human breast cancer cells, MCF‐7 and MDA‐MB‐231, were obtained from the Cell Bank of the Chinese Academy of Sciences (Shanghai, China). The cells were incubated in Roswell Park Memorial Institute‐1640 medium supplying with 10% fetal bovine serum (Gibco, Carlsbad, CA) and l‐glutamine (Invitrogen, Carlsbad, CA) at 37°C in a humidified atmosphere containing 5% CO_2_. The passage time of all cell lines was less than 3 months.

### 2.2 The quantitative reverse transcription‐polymerase chain reaction analysis

A detailed protocol of quantitative reverse transcription‐polymerase chain reaction (qRT‐PCR) analysis could be found in our previous publication.^38^ Quantitative RT‐PCR for mRNA was detected using an ABI 7500 real‐time PCR system and Absolute qPCR SYBR Green Mix (Applied Biosystems, Foster City, CA). The primer sequences used for *KIF11* mRNA detection were 5′‐GATGGACGTAAGGCAGCTCA‐3′ (forward) and 5′‐TGTGGTGTCGTACCTGTTGG‐3′ (reverse). *C*_t_ values for *KIF11* mRNA were normalized to β‐actin mRNA, which was used as internal controls. The $2^{-\Delta \Delta C_{t}}$ method was applied to calculate the relative expression of mRNA.

### 2.3 *KIF11* small‐hairpin RNA plasmids and transient transfectants construction

pGPU6/GFP/Neo was used to express small‐hairpin RNA (shRNA). pGPU6/GFP/Neo‐ *KIF11*‐Homo vectors were purchased from GenePharma (Shanghai). The target sequence of pGPU6/GFP/Neo‐*KIF11*‐404 was GCGGAAAGCTAGCGCCCATTC, and the target sequence of pGPU6/GFP/Neo‐*KIF11*‐1152 was GCTCGGGAAGCTGGAAATATA. MCF‐7 cells at 2 × 10^5^/well and MDA‐MB‐231 cells at 3 × 10^5^/well in a six‐well plates were transduced with the lipofection shRNAs and selected with 600 µg/mL G418 (BBL Life Science, Shanghai).

### 2.4 *miR‐30a* plasmids construction, transient transfection, and luciferase assay

To construct a plasmid containing the *KIF11* 3′‐UTR fused to the 3′‐end of the luciferase reporter, sequences containing the predicted *miR‐30a* target sites were synthesized and ligated into the pGLO‐control vector (Promega, Madison, WI). *KIF11* 3′‐UTR was amplified with the primers 5′‐AAACTAGCGGCCGCAATTTATATTCTTTTGTTTACAT‐3′ (forward) and 5′‐CTAGATGTAAACAAAAGAATATAAATTGCGGCCGCTAGTTT‐3′ (reverse) and was subcloned into a pGLO control vector with the restriction endonuclease XbaI site (italic font) to generate pGLO‐*KIF11*‐3′‐UTR. The 3′‐UTR of *KIF11* containing two putative *miR‐30a*‐binding sites was amplified and cloned into a pmirGLO‐control vector separately. In the mutated fragment, three mutational bases were introduced into the predicted *miR‐30a* target sites. Subsequently, cells were plated into 24‐well plates, cotransfected with the constructed plasmids, the plasmids with either *miR‐30a* or miR‐NC; were purchased from Vigene Bioscience (Rockville, MD). Collected cells and measured their luciferase activity after 48 hours using the Dual‐Luciferase Reporter Assay Kit (Promega, Madison, WI). The results are shown as the relative luciferase activity of the firefly/renilla ratio. All the transient transfections were performed using Lipofectamine 2000 (Invitrogen).

### 2.5 Cell proliferation analysis and drug treatment

Cell Counting Kit 8 (CCK8) was employed to determine the viable cells in cytotoxicity and proliferation assays. According to the manufacturer's instructions, we seeded 2500 cells per well for proliferation assay and 5000 cells/well for cytotoxicity test in a 96‐well plates. The incubation times for proliferation and cytotoxicity were 72 and 48 hours, respectively. Total 10 µL of the CCK8 reagent (Bimake, Houston, TX) directly added to each well, after incubating for 4 hours, reading the optical density (OD) at 450 nm with a BioTeK Synergy H1 plate reader (Winooski, VT). The value of OD 450 nm represents the number of viable cells.

### 2.6 Invasion assays

Details of the invasion assay were described in a previous publication.^39^ About 50 000 cells were seeded on the Matrigel (BD Biosciences, San Jose, CA) insert of the 24‐well chambers. After incubation for 72 hours in 5% CO_2_ at 37°C, the top of the Matrigel inserts were wiped with a cotton‐tipped swab to remove cells that had not migrated through the membrane. The cells on the lower surface of the membrane were stained with crystal violet solution and counted. Each experiment was performed three times.

### 2.7 Western blot analysis

Denatured total protein was extracted from breast cancer cells after transfected by *sh‐NC* or *sh‐KIF11*. Proteins separated by 10% sodium dodecyl sulphate‐polyacrylamide gel electrophoresis were transferred to polyvinylidene difluoride membranes. The membranes were blocked with 3% bovine serum albumin or 5% nonfat powdered milk in TBST for 2 hours at room temperature, then incubated with primary antibodies overnight at 4°C. The primary antibodies used were against KIF11 (Proteintech, Wuhan, China), E‐cadherin, extracellular‐signal‐regulated kinase (Erk), phospho Erk (p­Erk), protein kinase B (Akt), phosphorylated Akt (p­Akt), (all from Cell Signaling Technology, Danvers, MA), and glyceraldehyde 3‐phosphate dehydrogenase (GAPDH) (Sungene, Tianjin, China). After washing with TBST three times, the membranes were incubated with secondary antibodies conjugated with horseradish peroxidase‐­conjugated goat anti‐­rabbit IgG or goat anti‐­mouse IgG (ImmunoWay) for 1 hour at room temperature. After washing with TBST three times, the protein bands were measured by an Enhanced Chemiluminescence Kit (Beyotime) through a Clinx Science Instruments (Shanghai). The intensity of the specific bands was measured by Image J software.

### 2.8 Worldwide gene expression datasets

A total of 17 published microarray datasets containing survival information of breast cancer patients was downloaded from the Array Express database (www.ebi.ac.uk/arrayexpress) including GSE7390,^40^ GSE1456,^41^ GSE2034,^42^ GSE4922,^43^ GSE10885,^44^ GSE24450,^45^ GSE25066,^46^ GSE53031,^47^ GSE58812,^48^ GSE22220,^49^ GSE3143,^50^ GSE3494,^51^ GSE11121,^52^ GSE12276,^53^ GSE22226,^54^ GSE6532,^55^ and NKI^56^; two of The Cancer Genome Atlas‐Breast Invasive Carcinoma (TCGA‐BRCA) was downloaded from TCGA (https://www.cbioportal.org/). Datasets without prognostic outcome information were excluded. The clinical relevance and prognostic significance of *KIF11* in breast cancer were evaluated on the above datasets. Detailed information of the microarray datasets is summarized in Table S1.

The disease‐free survival (DFS) period was defined as the time from initial surgery until tumor recurrence, including local relapse and distant metastasis. The overall survival (OS) period was calculated as the time from initial surgery to the date when the patient was last seen. To normalize the mRNA expression levels among the above datasets, we restratified the scores of *KIF11* and other related genes into four grades (Q_1_, Q_2_, Q_3_, and Q_4_) based on the percentile for each independently downloaded dataset. For Cox analysis, less than the median was regarded as *KIF11*‐low (Q_1_ + Q_2_), while greater than or equal to the median was regarded as *KIF11*‐high (Q_3_ + Q_4_). The demographic distribution of *KIF11* is presented in Table S2.

### 2.9 Gene set enrichment analysis

To evaluate the correlations between *KIF11* expression and cancer‐related pathways, we conducted gene set enrichment analysis (GSEA) using the above microarray dataset GSE1456. The detailed protocol of GSEA was available on the Broad Institute GSEA website (www.broad.mit.edu/gsea) or from related references.^57^ Briefly, datasets and phenotype label files were created and loaded into GSEA software (v2.0.13). The gene sets were downloaded from the Gene Expression Omnibus (http://www.cancergenome.nih.gov/geo/). The phenotype label was *KIF11* expression. We set the number of permutations to 1000.

### 2.10 Target prediction and functional analysis of miRNA

The presumed target of *KIF11*‐related miRNA, especially the most significant *hsa*‐*miR‐30a*, we searched in microRNA.org (http://www.microrna.org/microrna/home.do). The above breast cancer microarray dataset GSE22220 was used to assess the role of *miR‐30a* and *KIF11* in breast cancer progression and prognosis.

### 2.11 Data management and statistical analysis

All data were analyzed using the SAS statistical software, version 9.2 (SAS Institute, Cary, NC), unless otherwise noted. The Student *t* test and one‐way analysis of variance were used for continuous data analyses, and the Pearson *χ* ^2^ test was used for categorical data analyses. We used Kaplan‐Meier survival analysis to draw the proportion of the population that was OS or DFS by follow‐up time in months. We calculated hazard ratios (HR) with 95% confidence intervals (CI) using Cox proportional hazards regression analysis to survey the association of *KIF11* expression levels with patient survival. Two‐sided *P* values less than .05 were considered statistically significant. Missing data were coded and excluded from the analysis.

## 3 RESULTS

### 3.1 Inhibition of *KIF11* causes growth blockage and invasion decrease in breast cancer cells

**Figure 1:**
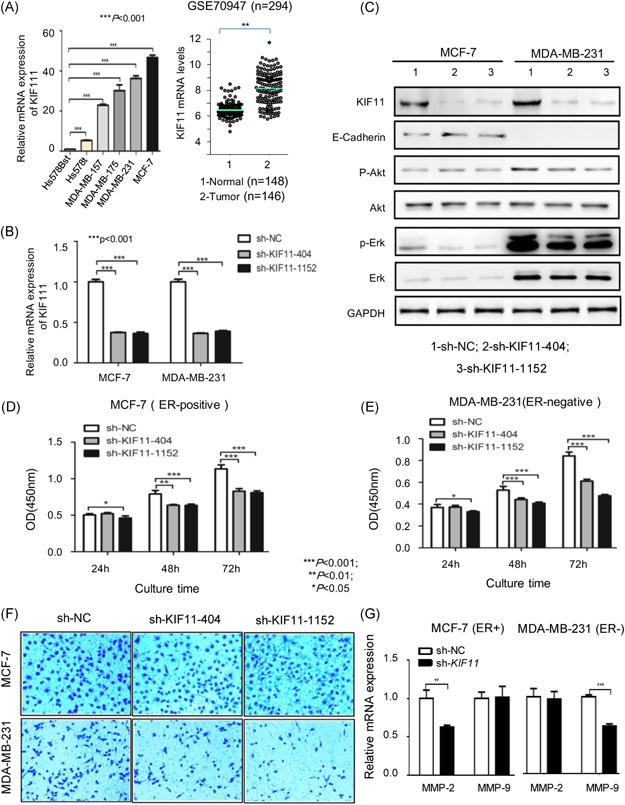
Inhibition of *KIF11* causes growth blockage, and invasion decrease in breast cancer cells. A, Expression of *KIF11* in cell lines and GSE70947 set. B, Relative mRNA expression of *KIF11* in MCF‐7 and MDA‐MB‐231 cells following transfection with *sh*‐*KIF11*, *sh‐NC* as control. C, The expression levels of KIF11, E‐cadherin, Akt, p‐Akt, Erk, and p‐Erk in transfectants were visualized by Western blot. D, The cell growth curve for MCF‐7 transfectants was measured with CCK8 assay. E, The cell growth curve for MDA‐MB‐231 transfectants was measured with CCK8 assay. F, The invasion chamber detected invasion ability in breast cancer cells. G, Expression levels of *MMP* were detected by qPCR. Shown were the representative results (mean ± standard deviation) of three independent experiments. Akt, protein kinase B; CCK8, Cell Counting Kit 8; ER, estrogen receptor; Erk, extracellular‐signal‐regulated kinase; GAPDH, glyceraldehyde 3‐phosphate dehydrogenase; *KIF11*, kinesin family member 11; MMP, matrix metalloproteinase; mRNA, messenger RNA; OD, optical density; p‐Akt, phosphorylated Akt; p‐Erk, phospho Erk; qPCR, quantitative polymerase chain reaction; sh‐NC, short hairpin negative control. *P < .05, **P < .01, ***P < .001 [Color figure can be viewed at wileyonlinelibrary.com]

To explore the role of *KIF11* in the development of breast cancer, we analyzed mRNA expression of *KIF11* in both cell lines and the GSE70947 dataset. Analysis results showed that mRNA expression levels of *KIF11* were significantly higher in tumor than in normal control (Figure 1A). To verify whether inhibition of *KIF11* could alleviate the development of breast cancer in vitro, the shRNA was used to downregulate the expression of *KIF11* in estrogen receptor (ER)‐positive breast cancer cell line MCF‐7 and ER‐negative breast cancer cell line MDA‐MB‐231. *sh‐NC* plasmids were employed as control. The qPCR results indicated that *sh‐KIF11* reduced the mRNA level of *KIF11* in both MCF‐7 and MDA‐MB‐231 (Figure 1B). After knocking down *KIF11* in MCF‐7 and MDA‐MB‐231, expression levels of KIF11, E‐cadherin, Akt, p‐Akt, Erk, and p‐Erk levels were measured. The levels of Akt, p‐Akt, and Erk did not change significantly after *KIF11* reduction, whereas p‐Erk levels slightly decreased in both MCF‐7 and MDA‐MB‐231, then E‐cadherin slightly increased in MCF‐7 but not expressed in MDA‐MB‐231 (Figure 1C). Inhibition of *KIF11* significantly delayed cell growth of both MCF‐7 and MDA‐MB‐231 (Figure 1D,E). The invasion assay also showed that *sh*‐*KIF11* could reduce invasive cells from 132.0 ± 12.4 per field (*sh‐NC*) to 92.5 ± 16.7 per field (*sh*‐*KIF11*‐404) and 83.5 ± 20.5 per field (*sh*‐*KIF11*‐1152) in MCF‐7 cells (*P* < .01), and from 118 ± 26.4 per field (*sh‐NC*) to 55.0 ± 20.4 per field (*sh*‐*KIF11*‐1152) in MDA‐MB‐231 cells (*P* < .05) (Figure 1F). Expression levels of *MMP* were significantly reduced (Figure 1G). These findings suggested that inhibition of *KIF11* could significantly inhibit the growth and invasive ability of both MCF‐7 (ER‐positive) and MDA‐MB‐231 (ER‐negative) cell lines.

### 3.2 *KIF11* is associated with poor differentiation and aggressive phenotypes of breast cancer

**Figure 2:**
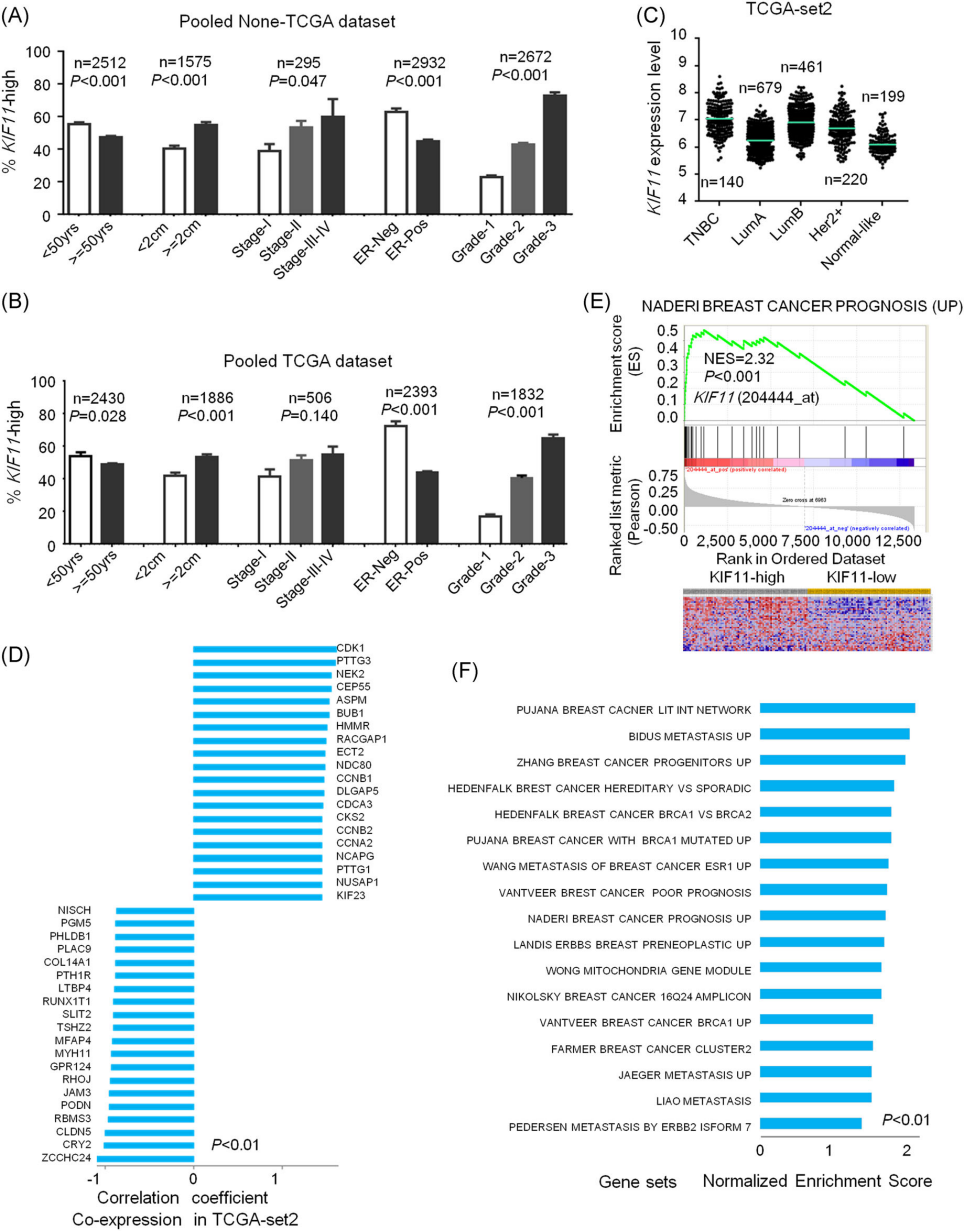
High expression of *KIF11* is related to invasiveness in breast cancer in downloaded datasets. A, The mRNA levels of *KIF11* and age, tumor size, ER status, and Elson grade of breast cancer in pooled dataset in non‐TCGA. Here, the *KIF11*‐high was defined as the *KIF11* mRNA level equal to or larger than median mRNA levels in each dataset. B, The mRNA levels of *KIF11* and age, tumor size, ER status, and Elson grade of breast cancer in pooled dataset in TCGA. C, The mRNA expression level of *KIF11* in different molecular subtypes of breast cancer in TCGA‐BRCA‐set2. D, Genes were coexpressed with *KIF11* in TCGA‐BRCA‐set2. E, Enriched gene signatures associated with poor prognosis were displayed. Heatmap depicts the expression levels of these genes. Red represents upregulation, and blue represents downregulation. F, NES of *KIF11* shown in a related pathway in ordered datasets. ER, estrogen receptor; HER2, human epidermal growth factor receptor 2; *KIF11*, kinesin family member 11; LumA, luminal A; LumB, luminal B; mRNA, messenger RNA; NES, normalized enrichment score; TCGA‐BRCA, The Cancer Genome Atlas‐Breast Invasive Carcinoma; TNBC, triple‐negative breast cancer [Color figure can be viewed at wileyonlinelibrary.com]

The expression data of *KIF11* could be obtained from all collected datasets to investigate the clinical relevance. Analysis results showed that mRNA expression levels of *KIF11* were significantly associated with the younger patient, lower ER levels, bigger tumor size, lymph node, and higher grade of breast cancer in none‐TCGA (Figure 2A) and TCGA datasets (Figure 2B). In TCGA‐BRCA‐set 2, mRNA expression levels of *KIF11* were significantly higher in aggressive molecular subtypes such as triple‐negative breast cancer (TNBC) than in normal‐like or LumA breast cancer (Figure 2C). The genes were coexpressed with *KIF11* in TCGA‐BRCA‐set 2, which including cyclin‐dependent kinase, abnormal spindle microtubule assembly, epithelial cell transforming, and mitotic checkpoint serine/threonine kinase were shown (Figure 2D). A further GSEA analysis indicated that high‐expression of *KIF11* significantly enriched in the gene signatures related to poor prognosis. The normalized enrichment score (NES) was 2.32 (Figure 2E). The correlation between *KIF11* and poor prognosis was further verified. Besides, more NES related to poor differentiation, metastasis, and so forth were indicated in breast cancer (Figure 2F). All of the above findings validated that mRNA levels of *KIF11* were significantly associated with aggressive phenotypes in breast cancer.

### 3.3 Prognostic significance of *KIF11* for breast cancers

**Figure 3:**
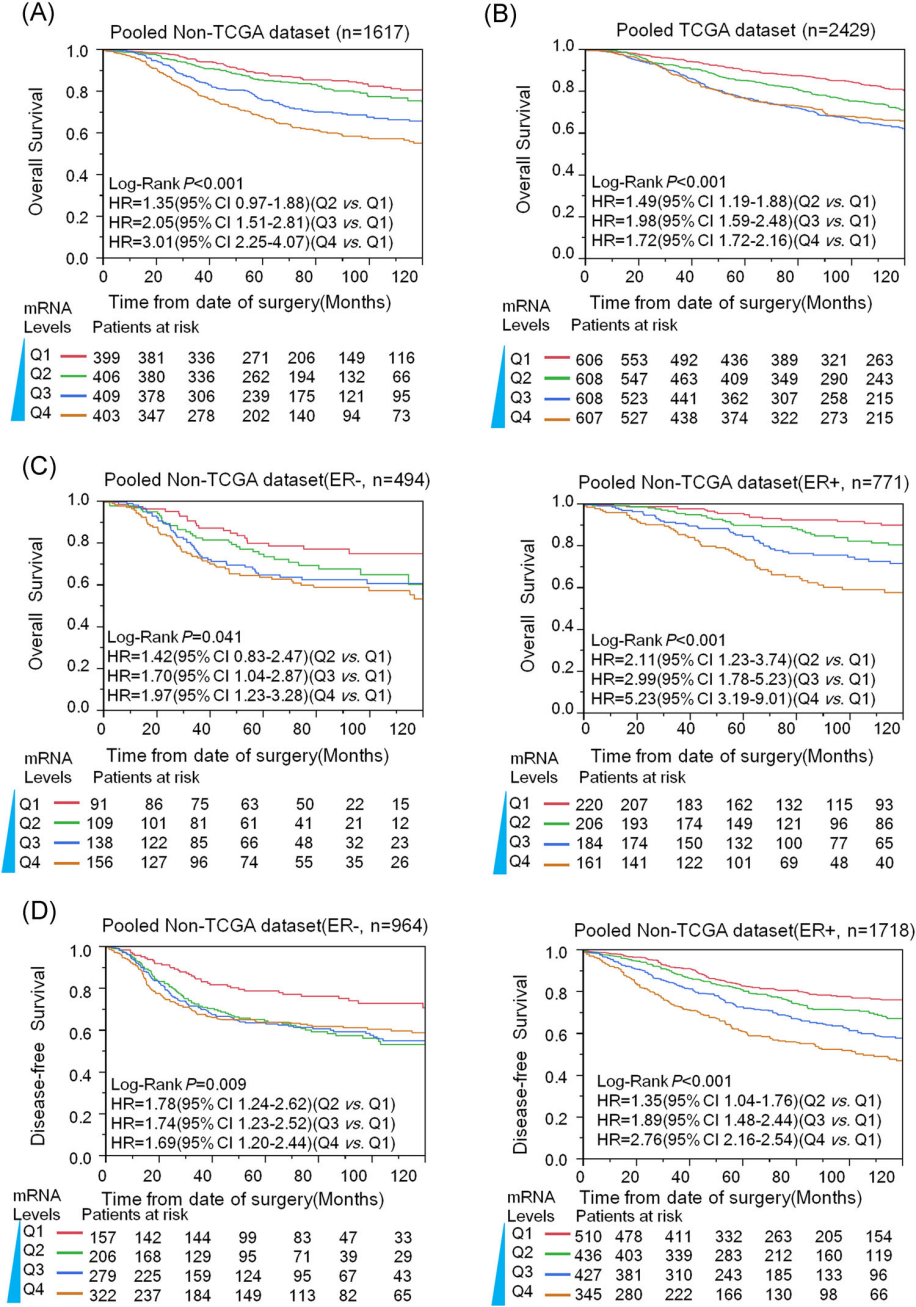
Kaplan‐Meier analysis was performed to investigate the *KIF11* and outcome of breast cancer among microarray datasets. We pooled all eligible breast cancers after normalizing. A, The overall survival (OS) analysis results of *KIF11* in pooled datasets in non‐TCGA. B, Analysis of *KIF11* for the OS in pooled datasets in TCGA. C, The prognostic performance of *KIF11* for the OS of both ER‐negative and ER‐positive breast cancers in non‐TCGA. D, Disease‐free survival results of *KIF11* from pooled datasets with disease‐free survival information in non‐TCGA. CI, confidence interval; ER, estrogen receptor; HR, hazard ratio; *KIF11*, kinesin family member 11; mRNA, messenger RNA; TCGA, The Cancer Genome Atlas [Color figure can be viewed at wileyonlinelibrary.com]

Since NES of *KIF11* was associated with poor prognosis, poor differentiation, and metastasis of breast cancer, the expression of *KIF11* could be related to poor outcomes in breast cancer. To address this hypothesis, we conducted a survival analysis on public microarray gene expression datasets. Here, we recategorized participants of each dataset into four subgroups (Q_1_, Q_2_, Q_3_, and Q_4_) according to the expression levels of *KIF11*. In Figure 3A, the mRNA levels of *KIF11* significantly impacted poor OS (left) of breast cancer in none‐TCGA datasets. Following increased *KIF11* levels, OS decreased in a dose‐dependent manner. The prognostic significance of *KIF11* was further analyzed in TCGA datasets (Figure 3B). ER‐negative breast cancers (including the HER2‐positive and TNBC subtypes) have a poor prognosis.^58^ Our results in Figure 2A,B, showed that *KIF11* had higher expression levels in ER‐negative breast cancers. Further stratified Kaplan‐Meier analysis with the pooled data explored that *KIF11* mRNA levels were significantly associated with poor OS (Figure 3C) and poor DFS (Figure 3D) in not only ER‐negative but also in ER‐positive breast cancers. It was confirmed that *KIF11* expression significantly impacted the poor survival of breast cancer.

Further survival analysis was conducted on every independent dataset by employing unique and multiple Cox proportional hazard analysis. The results are listed in Table 1. Q_1_ was the lowest expression subgroup, which was set as the relative point of reference. The HR of *KIF11* for OS and DFS mostly increased along with *KIF11* expression levels increased in none‐TCGA datasets. Particularly in higher *KIF11* levels (Q_4_), the significance almost could be observed in all datasets. The overall pooled analysis indicated that the HR of higher *KIF11* (Q_4_) for OS and DFS were 3.01 (95% CI: 2.25‐4.07) and 2.11 (95% CI: 1.78‐2.50), respectively. In TCGA datasets, the HR of *KIF11* for OS also increased along with *KIF11* expression levels increased, the HR of higher *KIF11* (Q_4_) for OS was 1.72 (95% CI: 1.38‐2.16).

**Table 1:** Uni‐ and multivariate analysis for *KIF11* and survival in microarray datasets

		Overall survival		Disease‐free survival	
		HR	Adjusted HR	HR	Adjusted HR
Dataset		(95% CI)	(95% CI)^a^	(95% CI)	(95% CI)^a^
GSE7390					
	Q_1_	Reference	Reference	Reference	Reference
	Q_2_	2.30 (0.97‐6.03)	2.24 (0.94‐5.92)	1.70 (0.94‐3.19)	1.64 (0.90‐3.09)
	Q_3_	1.97 (0.80‐5.24)	2.01 (0.78‐5.57)	1.33 (0.71‐2.54)	1.19 (0.60‐2.40)
	Q_4_	3.42 (1.52 ‐8.69)**	2.98 (1.23‐7.99)*	1.82 (0.99‐3.43)	1.65 (0.84‐3.31)
GSE1456					
	Q_1_	Reference	Reference	Reference	Reference
	Q_2_	3.74 (1.14‐16.69)*	4.45 (1.13‐29.38)*	2.51 (0.70‐11.65)	2.08 (0.57‐9.73)
	Q_3_	5.75 (1.89‐24.84)**	5.01 (1.18‐34.83)*	5.83 (1.92‐25.20)**	3.32 (0.93‐15.86)
	Q_4_	4.72 (1.50‐20.70)**	4.68 (1.02‐34.28)*	6.42 (2.12‐27.73)**	3.71 (0.99‐18.55)
GSE2034					
	Q_1_	N/A	N/A	Reference	Reference
	Q_2_			1.40 (0.77‐2.60)	1.40 (0.77‐2.61)
	Q_3_			1.82 (1.03‐3.33)*	1.85 (1.04‐3.38)*
	Q_4_			2.31 (1.31‐4.18)**	2.36 (1.33‐4.30)**
GSE4922					
	Q_1_	N/A	N/A	Reference	Reference
	Q_2_			1.67 (0.83‐3.52)	1.61 (0.79‐3.39)
	Q_3_			2.82 (1.47‐5.77)**	2.30 (1.16‐4.83)*
	Q_4_			3.48 (1.82‐7.09)**	2.65 (1.28‐5.76)**
GSE25066					
	Q_1_	N/A	N/A	Reference	Reference
	Q_2_			1.91 (1.08‐3.44)*	1.31 (0.71‐2.44)
	Q_3_			1.58 (0.93‐2.75)	1.16 (0.66‐2.08)
	Q_4_			1.52 (0.87‐2.71)	1.10 (0.61‐2.03)
GSE10885					
	Q_1_	Reference	Reference	Reference	Reference
	Q_2_	3.97 (1.18‐17.91)*	4.54 (1.28‐21.27)*	2.96 (1.15‐8.54)*	3.31 (1.19‐10.67)*
	Q_3_	5.14 (1.64‐22.52)**	4.53 (1.34‐20.82)*	3.84 (1.60‐10.62)**	3.91 (1.38‐12.99)**
	Q_4_	7.16 (2.35‐31.06)**	5.91 (1.59‐29.05)**	4.11 (1.70‐11.41)**	3.49 (1.12‐12.58)*
GSE22226					
	Q_1_	Reference	Reference	Reference	Reference
	Q_2_	0.55 (0.32‐0.94)*	0.57 (0.32‐1.01)	1.66 (0.67‐4.47)	1.52 (0.57‐4.48)
	Q_3_	0.55 (0.31‐0.95)*	0.57 (0.32‐1.03)	1.60 (0.61‐4.40)	1.23 (0.43‐3.76)
	Q_4_	0.56 (0.32‐1.00)*	0.57 (0.31‐1.06)	1.84 (0.73‐5.01)	1.52 (0.55‐4.57)
GSE58812					
	Q_1_	Reference	Reference	Reference	Reference
	Q_2_	0.56 (0.15‐1.84)	0.54 (0.14‐1.80)	1.63 (0.54‐5.41)	1.56 (0.52‐5.16)
	Q_3_	1.54 (0.59‐4.23)	1.92 (0.73‐5.32)	1.98 (0.68‐6.44)	2.43 (0.83‐7.98)
	Q_4_	1.11 (0.40‐3.17)	1.26 (0.45‐3.60)	1.83 (0.63‐5.96)	2.06 (0.71‐6.72)
GSE24550					
	Q_1_	Reference	N/A	Reference	N/A
	Q_2_	0.75 (0.48‐1.18)		0.98 (0.63‐1.52)	
	Q_3_	0.78 (0.49‐1.24)		0.93 (0.58‐1.47)	
	Q_4_	0.85 (0.52‐1.37)		0.96 (0.58‐1.57)	
GSE53031					
	Q_1_	N/A	N/A	Reference	Reference
	Q_2_			1.39 (0.83‐2.32)	1.41 (0.83‐2.38)
	Q_3_			1.15 (0.70‐1.92)	1.12 (0.66‐1.92)
	Q_4_			1.06 (0.64‐1.77)	1.08 (0.61‐1.90)
GSE3494					
	Q_1_	Reference	Reference	N/A	N/A
	Q_2_	1.62 (0.64‐4.39)	1.58 (0.59‐4.64)		
	Q_3_	2.46 (1.05‐6.40)*	2.24 (0.89‐6.40)*		
	Q_4_	3.96 (1.76‐10.07)**	3.22 (1.25‐9.46)		
GSE6532					
	Q_1_			Reference	Reference
	Q_2_			0.97 (0.64‐1.48)	1.01 (0.52‐1.64)
	Q_3_			0.36 (0.22‐0.56)**	0.52 (0.30‐0.87)*
	Q_4_			0.20 (0.12‐0.34)*	0.39 (0.20‐0.72)*
GSE11121					
	Q_1_			Reference	Reference
	Q_2_			1.69 (0.66‐4.58)	1.44 (0.56‐3.95)
	Q_3_			1.49 (0.59‐4.06)	1.13 (0.43‐3.21)
	Q_4_			2.90 (1.24‐7.54)*	1.95 (0.75‐5.53)
GSE12276					
	Q_1_			Reference	N/A
	Q_2_			1.34 (0.91‐1.99)	
	Q_3_			1.71 (1.15‐2.55)**	
	Q_4_			1.40 (0.94‐2.07)	
NKI set					
	Q_1_	Reference	Reference	Reference	Reference
	Q_2_	4.60 (2.09‐11.53)**	3.12 (1.40‐7.92)**	2.78 (1.55‐5.20)**	2.35 (1.30‐4.41)**
	Q_3_	4.37 (1.96‐11.04)**	2.38 (1.03‐6.19)*	3.22 (1.81‐5.98)**	2.41 (1.40‐4.22)**
	Q_4_	4.80 (2.17‐12.11)**	1.83 (0.75‐4.96)	2.95 (1.65‐5.52)**	1.89 (0.98‐3.76)
Pooled GEO dataset					
	Q_1_	Reference	Reference	Reference	Reference
	Q_2_	1.35 (0.97‐1.88)	1.63 (1.05‐2.59)*	1.41 (1.18‐1.69)**	1.47 (1.15‐1.88)**
	Q_3_	2.05 (1.51‐2.81)**	1.80 (1.16‐2.85)**	1.69 (1.42‐2.01)**	1.66 (1.31‐2.12)**
	Q_4_	3.01 (2.25‐4.07)**	2.69 (1.75‐4.24)**	2.11 (1.78‐2.50)**	1.95 (1.53‐2.49)**
TCGA‐BRCA‐set1					
	Q_1_	Reference	Reference	Reference	Reference
	Q_2_	0.93 (0.50‐1.71)	1.07 (0.57‐1.98)	1.05 (0.49‐2.25)	1.15 (0.53‐2.51)
	Q_3_	1.19 (0.66‐2.14)	1.41 (0.76‐2.59)	1.12 (0.52‐2.41)	1.16 (0.52‐2.58)
	Q_4_	0.81 (0.43‐1.48)	0.88 (0.43‐1.75)	1.27 (0.63‐2.63)	1.14 (0.52‐2.52)
TCGA‐BRCA‐set2					
	Q_1_	Reference	Reference	NA	NA
	Q_2_	1.62 (1.26‐2.08)**	1.50 (1.16‐1.94)**		
	Q_3_	2.16 (1.70‐2.75)**	1.75 (1.37‐2.25)**		
	Q_4_	1.92 (1.51‐2.46)**	1.38 (1.07‐1.80)*		
Pooled TCGA dataset					
	Q_1_	Reference	Reference	Reference	Reference
	Q_2_	1.49 (1.19‐1.88)**	1.52 (1.18‐1.97)**	1.05 (0.63‐2.84)	1.14 (0.52‐2.48)
	Q_3_	1.98 (1.59‐2.48)**	1.73 (1.35‐2.24)**	1.12 (0.52‐2.41)	1.22 (0.55‐2.60)
	Q_4_	1.72 (1.38‐2.16)**	1.39 (1.07‐1.82)*	1.27 (0.63‐2.63)	1.15 (0.53‐2.56)

*Note*: Uni‐ and multivariate analysis were conducted to evaluate HR of *KIF11*.

Abbreviations: CI, confidence interval; ER, estrogen receptor; GEO, Gene Expression Omnibus; HR, hazard ratio; *KIF11*, kinesin family member 11; NA, not applicable; TCGA‐BRCA, The Cancer Genome Atlas‐Breast Invasive Carcinoma.

^a^ For multivariate analysis, HR was adjusted by age, ER status, Elston grade in the GSE7390, GSE4922, GSE25066, GSE10885, GSE2226, GSE53031, GSE3494, GSE3494, NKI set, TCGA‐BRCA‐set2, pooled GEO analysis, and in pooled TCGA analysis. In the GSE1456, HR was adjusted by Elston grade and ER; in the GSE2034, HR was adjusted by ER status; in the GSE58812 and TCGA‐BRCA‐set1, HR was adjusted by age and ER status; and in the GSE11121, HR was adjusted by grade.

* Statistical significance, *P* < .05.

** Statistical significance, *P* < .01.

### 3.4 Reduction of *KIF11* sensitizes chemotherapy and radiotherapy in breast cancer

**Figure 4:**
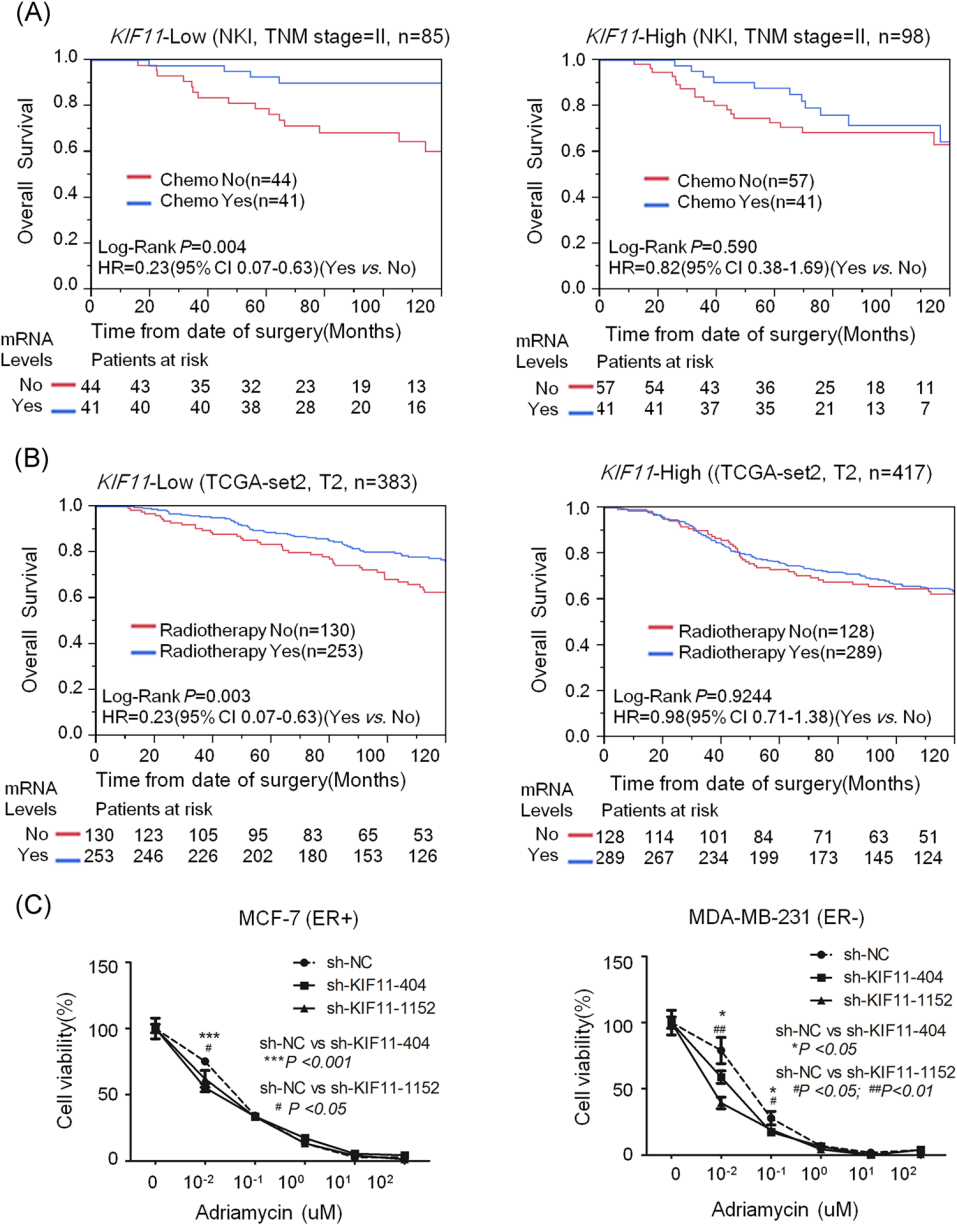
Reduction of *KIF11* expression might enhance chemo‐ and radiotherapy efficiency in breast cancers. A, The stratification analysis was conducted on stage II breast cancer patients to compare the chemotherapy efficacy between *KIF11*‐high expression and *KIF11*‐low expression in the NKI set. The OS of breast cancer patients was shown. B, The stratification analysis was conducted on T2 breast cancer patients to compare the radiotherapy efficacy between *KIF11‐*high expression and *KIF11‐*low expression in TCGA‐BRCA‐set2. The OS of breast cancer patients was shown. C, Reduction of *KIF11* expression might enhance the cytotoxicity of adriamycin in breast cancers. About 5000 cells (MCF‐7 or MDA‐MB‐231) per well were seeded on 96‐cell plates and transfected with *sh‐NC, sh‐KIF11*. After transfection, cancer cells were treated with 0, 0.01, 0.1, 1, 10, and 100 µM of adriamycin for 48 hours. Then, the cytotoxicity was measured by CCK8 assay. Shown were the representative results (mean ± standard deviation) of three independent experiments. CCK8, Cell Counting Kit 8; CI, confidence interval; ER, estrogen receptor; HR, hazard ratio; *KIF11*, kinesin family member 11; mRNA, messenger RNA; OS, overall survival; sh‐NC, short hairpin negative control; TCGA‐BRCA, The Cancer Genome Atlas‐Breast Invasive Carcinoma; TNM, tumor, node, metastasis [Color figure can be viewed at wileyonlinelibrary.com]

Chemotherapy is usually performed to patients with advanced stages of breast cancer (stage III or IV). For stage II breast cancer, the application of chemotherapy is determined by tumor size, grade, and other indicators. In these patients, a therapeutic biomarker would be beneficial for selecting chemotherapy. Our population study demonstrated that chemotherapy significantly improved the OS of stage II breast cancer patients with *KIF11*‐low expression in NKI dataset (logrank *P* = .004; HR = 0.23; 95% CI, 0.07‐0.63) rather than with the *KIF11*‐high expression (logrank *P* = .59; HR = 0.82; 95% CI, 0.38‐1.69) (Figure 4A). In TCGA‐BRCA‐set 2, radiotherapy significantly improved the OS of T2 breast cancer patients with *KIF11*‐low expression but not with the *KIF11*‐high expression (Figure 4B). Inhibition of *KIF11* by shRNA could improve the efficacy of adriamycin on breast cancer cells (Figure 4C). These findings suggest that the silence of *KIF11* can significantly enhance the effects of chemotherapy and radiotherapy in breast cancer. *KIF11* can also be employed as a therapeutic target and can serve as a biomarker for selecting chemotherapy and radiotherapy in breast cancer treatment.

### 3.5 Screening of *KIF11*‐targeting *miR‐30a*

**Figure 5:**
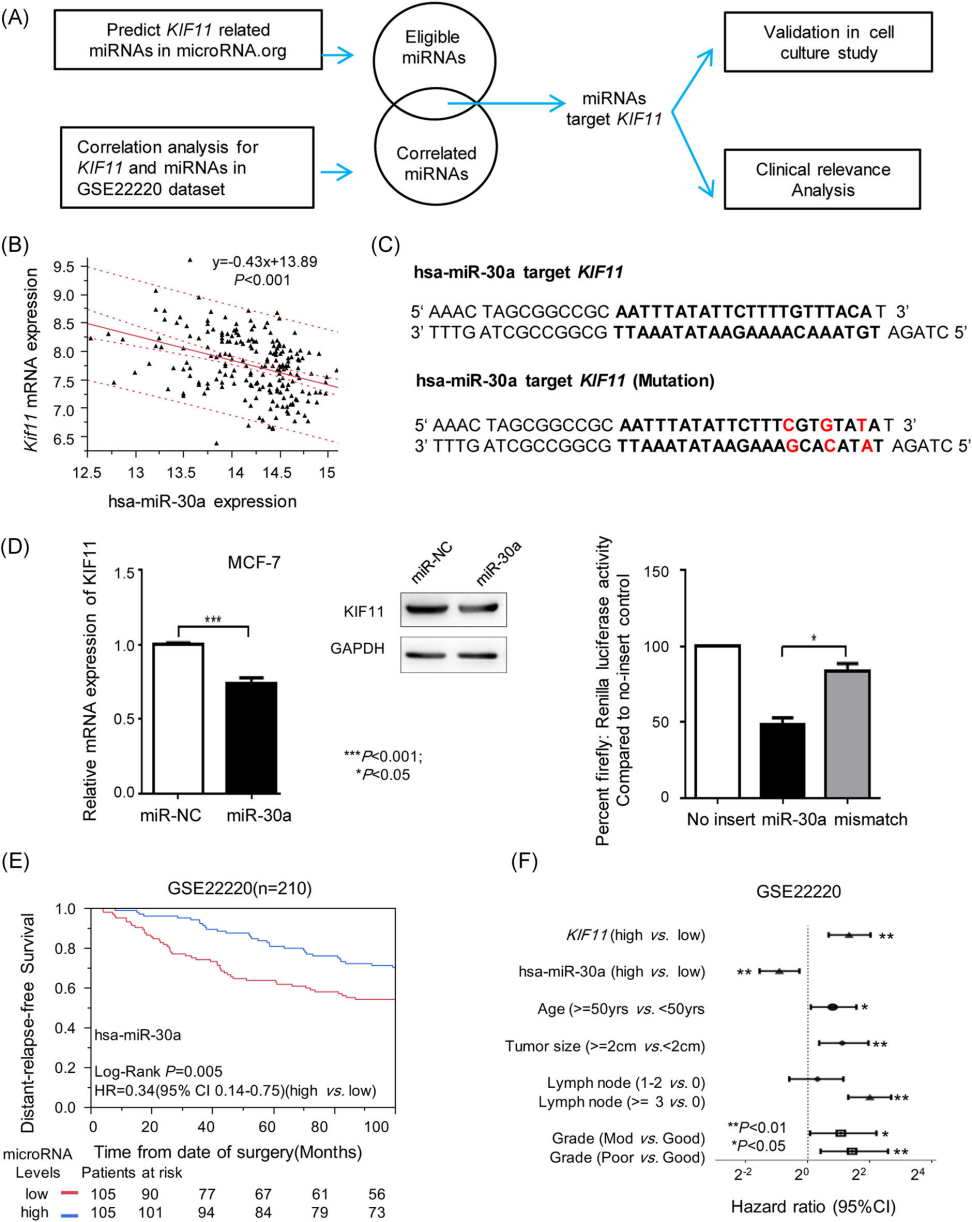
Correlated analysis of *KIF11* and *hsa*‐*miR‐30a*. A, The flowchart of the analysis and function validation about *KIF11*‐related miRNAs. B, Linear regression analysis between *KIF11* mRNA expression and *hsa‐miR‐30a* expression in the GSE22220 dataset. C, The sequence of *hsa‐miR‐30a* target *KIF11* was shown. D, Relative mRNA expression and protein expression of *KIF11* after *miR‐30a* transfection (left). Luciferase assay (right). E, Kaplan‐Meier distant‐relapse‐free survival analysis of *hsa‐miR‐30a* in the GSE22220. F, Comparison of prognostic performance of *KIF11* and *hsa‐miR‐30a* with tumor size, lymph node involvement, and Elston grade in breast cancer. CI, confidence interval; GAPDH, glyceraldehyde 3‐phosphate dehydrogenase; HR, hazard ratio; miRNA, microRNAs; mRNA, messenger RNA; *KIF11*, kinesin family member 11. *P < .05, **P < .01, ***P < .001 [Color figure can be viewed at wileyonlinelibrary.com]

miRNA is an essential transcriptional regulator involved in the various cancerous process. Here, we screened out eligible target miRNAs using both predicting *KIF11*‐related miRNAs in microRNA.org and correlation analysis for *KIF11* and miRNAs in the GSE22220 dataset (Figure 5A), to validate in cell culture study and to conduct clinical relevance analysis. *Hsa‐miR‐30a* expression was negatively correlated with *KIF11* mRNA expression (Figure 5B). The sequence of *hsa‐miR‐30a* target *KIF11* was shown in Figure 5C. *miR‐30a* transfection inhibited *KIF11* mRNA and protein expression (Figure 5D, left), luciferase assay further verified inhibited effects of *miR‐30a* to *KIF11* (Figure 5D, right). Analysis results based on the GSE22220 set showed that expression levels of *hsa*‐*miR‐30a* were significantly positively correlated with disease‐relapse‐free survival of breast cancer (Figure 5E). The prognostic performance of *KIF11* and *miR‐30a* could be compared with age, tumor size, and grade. *KIF11* and *hsa*‐*miR‐30a* had better prognostic capabilities than lymph node involvement (Figure 5F). The above findings suggest that *KIF11* and *miR‐30a* could serve as a prognostic biomarker to predict poor outcome in breast cancers, and *miR‐30a* in breast cancer could suppress the expression of *KIF11*.

## 4 DISCUSSION

In this study, we demonstrated that *KIF11* is highly expressed in breast cancer cell lines and closely related to poor differentiation and aggressive phenotypes in breast cancer patients. By GSEA analysis, we verified that *KIF11* enrichment occurs in breast cancer. Our data showed that *KIF11* mRNA levels were consistent with patient prognosis through pooled dataset analysis. Moreover, our data further indicate that high expression of *KIF11* may compromise chemo‐ and radiotherapy efficiency.

Previous studies have identified the potential role of kinesins in the breast cancer cell. Elevated expression levels of kinesins *KIF14*, *KIF4A*, *KIF20A*, and *KIF23*, *KIF2C*, and *KIFC1* have been reported in breast cancer cell lines, other kinesins *KIF10*, *KIF18A*, and *KIF15* have been linked to prognostic indicators in breast cancer patients.^59^ One study found that *KIF26B* is independently linked to the prognostic outcome in breast cancer.^60^ *KIF11* was found overexpressed in human pancreatic cancer samples and promoted cell proliferation in an ATPase activity‐dependent manner, leading to the accumulation of polyploid cells.^17^ Another research showed that overexpression of *KIF11* was related to poor differentiation of bladder cancer.^18^ Inhibition of *KIF11* with a highly specific small‐molecule inhibitor has been proven to delay the growth of commonly treatment‐resistant glioblastoma tumor cells and to hamper tumor initiation.^20^ *KIF11* increasingly expressed in high stage and malignant tumor cells.^23^ *KIF11* expression has also predicted treatment response with platinum chemotherapy in patients with advanced NSCLC. Here, downregulation of *KIF11* suppresses cell proliferation, invasion, and chemoresistance in MCF‐7 and MDA‐MB‐231 cells.

Furthermore, our validation in vitro study and clinical relevance analysis suggest that *miR‐30a* could be a negative regulator of *KIF11* in breast cancer development. miRNAs have proven to be a distinguished group of ribonucleotides that play a crucial role in human cancer. miRNA expression profiles have been proposed as a method to classify tumor stages and prognosis.^61^, ^62^, ^63^, ^64^ A recent review highlighted the predictive value of miRNAs in breast cancer patients before chemotherapy, radiotherapy, or surgical intervention.^65^ Researchers have discussed the potential role of miRNAs in breast cancer management, particularly in improving current prognosis and achieving individualized cancer care. On one side, miRNAs can function as oncogenes via negative inhibition of tumor suppressor genes; on the other side, induction of tumor suppressor miRNAs may result in the prevention or treatment of breast tumors. Further investigation of the functional roles of miRNAs would help us in gathering more knowledge of carcinogenesis, tumor biomarkers, and therapeutic drug discovery.^66^

Expression of *KIF11* and *miR‐30a* is associated with the development and outcome of breast cancer. First, in vitro assays with *KIF11* knockdown significantly inhibited cell proliferation and invasion. Second, the OS and DFS in breast cancer databases were significantly lower in high‐*KIF11* breast cancer than in low‐*KIF11* breast cancer. Third, the expression between *KIF11* and *miR‐30a* shared a negative correlation (Figure 5B). Given these findings, we propose that *KIF11* contributes to the development of breast cancer, and *miR‐30a* suppresses the *KIF11* expression. Undoubtedly, extensive investigations are required to illuminate the elaborate mechanism of *KIF11* in the development and regulation of breast cancer, and in‐depth studies are also needed to uncover the interactions between *KIF11* and *miR‐30a*.

Taken together, we demonstrate a critical role of *KIF11* in promoting invasion and predicting poor prognosis in breast cancer patients. The levels between *KIF11* and *miR‐30a* present a significantly negative correlation in breast cancer databases. Our findings highlight that *miR‐30a* and *KIF11* could be employed as promising prognostic biomarkers and therapeutic targets for breast cancers.

## CONFLICT OF INTERESTS

The authors declare that there are no conflict of interests.

## Supporting information

Supporting information

Supporting information

## Data Availability

The data that support the findings of this study are available from the corresponding author upon reasonable request.
