# Supplementary material for: Kinesin family member 11 is a potential therapeutic target and is suppressed by microRNA‐30a in breast cancer
Source: Mol Carcinog. 2020 Apr 29;59(8):908–22. doi: 10.1002/mc.23203 (PMC7384136; doi:10.1002/mc.23203)
Supplement: Supplementary file 1 — Supporting information [file MC-59-908-s001.doc]

**Table S1.** Summary of world-wide breast cancer gene expression datasets

| Accession No. | Assessable  cases | Date of study | Platforms* | Age at diagnosis | Country | Reference# |
| --- | --- | --- | --- | --- | --- | --- |
| GSE1456 | 159 | 1994-1996 | GPL96, GPL97 | N/A | Sweden | 41 |
| GSE2034 | 286 | 1980-1995 | GPL96 | N/A | USA | 42 |
| GSE4922 | 289 | 1987-1989 | GPL96, GPL97 | 63(28-93) | Singapore | 43 |
| GSE53031 | 167 | 1996-2010 | GPL13667 | 36(29-47) | Belgium | 47 |
| GSE7390 | 198 | 1980-1998 | GPL96 | 46(24-60) | Canada | 40 |
| GSE10885 | 153 | N/A | GPL885, GPL887 | 55(24-88) | USA | 44 |
| GSE22220 | 216 | N/A | GLP6098 | 55(26-80) | UK | 49 |
| GSE22226 | 129 | N/A | GPL1708,GPL4133 | 48(31-65) | USA | 54 |
| GSE24450 | 183 | 1997-2004 | GPL6947 | N/A | Finland | 45 |
| GSE25066 | 508 | N/A | GLP96 | 49(24-72) | USA | 46 |
| GSE58812 | 107 | 1998-2007 | GPL570 | 57(28-84) | France | 48 |
| GSE3143 | 158 | N/A | GPL8300 | N/A | USA | 50 |
| GSE3494 | 251 | 1987-1989 | GPL96, GPL97 | 64(28-93) | Singapore | 51 |
| GSE11121 | 200 | 1988-1998 | GPL96 | N/A | Germany | 52 |
| GSE12276 | 204 | N/A | GPL570 | N/A | Netherlands | 53 |
| GSE6532 | 244 | N/A | GPL96, GPL97, GPL570 | 59(32-88) | Canada | 55 |
| NKI# | 295 | 1984-1995 | Agilent 25K Chip | 44(26-53) | Netherlands | 56 |
| TCGA BRCA-set1 | 526 | N/A | N/A | 58(26-90) | N/A |  |
| TCGA BRCA-set2 | 1094 | N/A | N/A | 62(22-96) | N/A |  |

* Platforms:

GPL96: [HG-U133A] Affymetrix Human Genome U133A Array; GPL97: [HG-U133B] Affymetrix Human Genome U133B Array; GPL570: [HG-U133_Plus_2] Affymetrix Human Genome U133 Plus 2.0 Array; GPL13667: [HG-U219] Affymetrix Human Genome U219 Array; GPL8269: Agilent UNC PerouLab 244K Custom Human Array version 5; GPL1708: Agilent-012391 Whole Human Genome Oligo Microarray G4112A; GPL4133: Agilent-014850 Whole Human Genome Microarray 4x44K G4112F; GPL885: Agilent-011521 Human 1A Microarray G4110A; GPL887: Agilent-012097 Human 1A Microarray (V2) G4110B; GPL6947: Illumina HumanHT-12 V3.0 expression beadchip. GPL6098: Illumina humanRef-8 v1.0 expression beadchip; GPL8178:Illumina Human v1 MicroRNA expression beadchip; GPL8300: [HG-U95Av2] Affymetrix Human Genome U95 Version 2 Array
