# Supplementary material for: Kinesin family member 11 is a potential therapeutic target and is suppressed by microRNA‐30a in breast cancer
Source: Mol Carcinog. 2020 Apr 29;59(8):908–22. doi: 10.1002/mc.23203 (PMC7384136; doi:10.1002/mc.23203)
Supplement: Supplementary file 2 — Supporting information [file MC-59-908-s002.doc]

Table S2. Demographic distribution of KIF11 in pooled None-TCGA datasets and pooled TCGA datasets
	Pooled GEO datasets	Pooled TCGA datasets	
	High(%*)	Low(%*)	p value†	High(%*)	Low(%*)	p value†	
Age							
<50yrs	669(55.6)	535(44.4)		256(45.9)	256(45.9)		
≥50yrs	618(47.3)	690(52.7)	<0.001	958(51.2)	958(51.2)	0.028	
Elson histological grade							
1=Well	98(22.9)	330(77.1)		28(17.0)	137(83.0)		
2=Mod	534(42.9)	712(57.1)		301(40.6)	440(59.4)		
3=Poor	726(72.8)	272(27.2)	<0.001	603(65.1)	323(34.9)	<0.001	
ER status							
Negative	630(63.1)	368(36.9)		395(72.3)	151(27.7)		
Positive	871(45.0)	1063(55.0)	<0.001	809(43.8)	1038(56.2)	<0.001	
PR status							
Negative	217(66.4)	250(33.6)		670(62.6)	401(37.4)		
Positive	250(46.6)	286(53.4)	<0.001	542(40.1)	809(59.9)	<0.001	
Tumor size							
<2mm	237(40.6)	346(59.4)		250(42.2)	343(57.8)		
≥2mm	546(55.0)	446(45.0)	<0.001	694(53.7)	599(46.3)	<0.001	
Lymph node status							
Negative	650(47.7)	713(52.3)		126(49.4)	129(50.6)		
Positive	455(57.7)	334(42.3)	<0.001	134(51.5)	126(48.5)	0.629	
Molecular subtype							
Luminal A	103(27.0)	279(73.0)		159(23.4)	520(76.6)		
Luminal B	177(68.6)	81(31.4)		368(79.8)	93(20.2)		
HER2 +	106(67.1)	52(32.9)		139(63.2)	81(36.8)		
TNBC	351(68.4)	162(31.6)		160(80.4)	39(19.6)		
Normal-like	41(23.8)	131(76.2)	<0.001	18(12.9)	122(87.1)	<0.001	
TNM stage							
I	32(39.0)	50(61.0)		37(41.6)	52(58.4)		
II	98(53.6)	85(46.4)		152(51.5)	143(48.5)		
III-IV	18(60.0)	12(40.0)	0.047	67(54.9)	55(45.1)	0.140	
Tumor stage							
T1				51(38.6)	81(61.4)		
T2				202(54.3)	170(45.7)		
T3-T4				9(45.0)	11(55.0)	0.007	
Chemotherapy adjuvant							
No	177(57.3)	132(42.7)		703(43.8)	805(53.4)		
Yes	157(43.9)	201(56.1)	0.001	250(63.1)	146(36.9)	<0.001	
Hormone therapy							
No	127(49.8)	128(50.2)		381(52.2)	349(47.8)		
Yes	21(52.5)	19(47.5)	0.751	572(48.7)	602(51.3)	0.141	
Radio therapy							
No				357(46.5)	410(53.5)		
Yes				596(52.4)	47.6(47.6)	0.012	
Note: There are 2512, 2672, 2932, 863,1575, 2152, 1483, 295, 667 and 295 cases in Age, Elson histological grade, ER status, PR status, Tumor size, Lymph node status, Molecular subtype, TNM stage, Chemotherapy adjuvant and Hormone therapy in pooled GEO datasets;
There are 2430, 1832, 1886, 2393, 2422, 1699, 515, 506, 506,1904, 1904 and 1904 missing cases in Age, Elson histological grade, Tumor size, ER status, PR status , Molecular type ,Lymph node status, TNM stage, Tumor stage, Chemotherapy adjuvant, Hormone therapy, Ratio therapy.
* % represent positive rate of Kif11-High it equal to N High/(NHigh + NLow)×100%.
† p values was based on Pearson Chi square test.  
